# Supplementary material for: Activity-induced instabilities of brain organoids
Source: Eur Phys J E Soft Matter. 2021 Dec 7;44(12):147. doi: 10.1140/epje/s10189-021-00149-z (PMC8651611; doi:10.1140/epje/s10189-021-00149-z)
Supplement: Supplementary file 1 — Supplementary material 1 (pdf 226 KB) [file 10189_2021_149_MOESM1_ESM.pdf]

I. NUMERICAL MODEL

We model the brain organoid as a biphasic active nematic system [1]. This allows us to use the established active nematohydrodynamical equations, where we solve for the velocity $\boldsymbol{v}$, density ρ, orientational order $\boldsymbol{Q}$, and a binary order parameter ϕ. Four coupled equations detailed below describe the time evolution of the continuous fields.

The active gel region represents the cortex, surrounding a passive isotropic cavity, which represents the lumen. We differentiate between the cortex and lumen regions by using a binary order parameter ϕ, with $\phi \sim 1$ in the cortex and $\phi \sim 0$ within the lumen region. The binary order parameter is evolved according to a Cahn-Hilliard equation [2]

$$\partial_t \phi + \partial_k (v_k \phi) = \mu, \tag{1}$$

where

$$\mu = \Gamma_\phi \left(\frac{\delta \mathcal{F}}{\delta \phi} - \partial_k \left(\frac{\delta \mathcal{F}}{\partial_k \delta \phi} \right) \right) \tag{2}$$

is the chemical potential, which is calculated from the functional derivative of a free energy $\mathcal{F}$ (detailed below) with respect to ϕ and Γ_ϕ is the mobility coefficient that sets the rate of relaxation towards the free energy minimum.

Within the cortex region, the cells and their corresponding cytoskeletons are extended radially with clear orientational order. To account for the orientational order associated with this micro-structural feature of the cortex, we introduce a nematic order parameter that is a symmetric, traceless tensor $Q_{\alpha\beta} = S\left(q_\alpha q_\beta - \frac{1}{2}\delta_{\alpha\beta}\right)$ [3], where S represents the magnitude of the orientational order, and q indicates its direction. The orientational order parameter evolves according to the Beris-Edwards equation [4, 5]

$$(\partial_t + v_k \partial_k) Q_{ij} - \tilde{S}_{ij} = H_{ij}, \tag{3}$$

where $\boldsymbol{v}$ denotes fluid velocity. The response of the orientation field to velocity gradients is accounted for by the co-rotation term

$$\tilde{S}_{ij} = \xi E_{ij} + Q_{ik}\Omega_{kj} - \Omega_{ik}Q_{kj}, \tag{4}$$

with the strain rate tensor $E_{ij} = (\partial_i v_j + \partial_j v_i)/2$ and the vorticity tensor $\Omega_{ij} = (\partial_i v_j - \partial_j v_i)/2$ describing the symmetric and asymmetric parts of the velocity gradient tensor, respectively. In addition, ξ denotes a flow-alignment parameter, which determines the collective response of the orientation field to gradients in the velocity field. The advection and co-rotation dynamics (lhs of eq. 3) are subsequently supplemented by relaxation of the orientational order toward thermodynamic equilibrium, described by the molecular field

$$H_{ij} = -\Gamma \frac{\delta \mathcal{F}}{\delta Q_{ij}}, \tag{5}$$

which is calculated from the functional derivative of the free energy with respect to the orientational order parameter Q_{ij}. Γ indicates the rotational diffusion coefficient, which also sets the rate of relaxation towards the free energy minimum.

The total free energy of the system includes contributions from the Landau-De Gennes free energy for the bulk nematic (f_Q), and Frank free energy for orientational deformation ($f_{\nabla Q}$) terms, as well as a bulk free energy of the binary mixture (f_ϕ) and an interfacial term ($f_{\nabla\phi}$); $\mathcal{F}[\boldsymbol{Q}, \phi] = \int d^2\boldsymbol{r}\,(f_Q + f_{\nabla Q} + f_\phi + f_{\nabla\phi})$, with

$$f_Q = \tfrac{1}{2}\mathcal{C}\left(\phi S_n - 2Q_{ij}Q_{ij}\right)^2, \tag{6}$$

$$f_{\nabla Q} = \tfrac{1}{2}L \partial_k Q_{ij} \partial_k Q_{ij}, \tag{7}$$

$$f_\phi = \tfrac{1}{2}\mathcal{A}\phi^2 (1-\phi)^2, \tag{8}$$

$$f_{\nabla\phi} = \tfrac{1}{2}K \partial_k \phi \partial_k \phi. \tag{9}$$

The nematic free energy part consists of a Landau-De Gennes contribution f_Q which accounts for the coupling between the binary and orientational order parameters, which favors S to be equal to an equilibrium value S_n in $\phi = 1$ regions and a Frank-Oseen elastic energy term $f_{\nabla Q}$ that penalizes orientational deformations. The last two terms include a double-well potential f_ϕ to ensure phase-separation into two phases of $\phi = 1$ and $\phi = 0$, and an interface term $f_{\nabla\phi}$ that penalizes gradients in binary order parameter giving rise to surface tension, as detailed in sect. II below.

The evolution of the binary and orientational order parameters are subsequently completed by means of a coupling to the Navier-Stokes equations

$$\begin{aligned} \partial_i v_i &= 0, \\ \rho\left(\partial_t + v_k \partial_k\right) v_i &= \partial_j \Pi_{ij}, \end{aligned} \tag{10}$$

where Π_{ij} describes the stress tensor containing both a viscous contribution $\Pi_{ij}^{viscous} = 2\eta E_{ij}$, and an elastic contribution $\Pi_{ij}^{elastic} = -P\delta_{ij} - \lambda_\xi S H_{ij} + Q_{ik}H_{kj} - H_{ik}Q_{kj}$. Here η denotes fluid viscosity and P denotes the isotropic pressure. In addition, capillary stresses $\mathbf{\Pi}^{\text{cap}} = (\mathcal{F} - \mu\phi)\boldsymbol{I} - \boldsymbol{\nabla}\phi\left(\frac{\delta\mathcal{F}}{\delta\boldsymbol{\nabla}\phi}\right)$ account for the contributions from variations in the binary order parameter ϕ at the interface between the active and passive phases. Importantly, in addition to the dissipative stresses, we also account for the effect of active stresses generated within the cortex. To this end, we introduce an active stress in the form of coarse-grained dipoles acting along the orientation field [6]. We assume a uniform spread of dipoles throughout the organoid, resulting in uniform active stress throughout the active annulus. This leads to an additional *active* contribution to the stress tensor [6, 7]

$$\Pi_{ij}^{active} = \alpha Q_{ij}, \tag{11}$$

with α a proportionality constant scaling with activity. The sign of α determines the nature of active stresses, with $\alpha > 0$ for contractile and $\alpha < 0$ for extensile activities. Due to the contractility of the stem cells we use $\alpha > 0$ in the case of the model brain organoid considered here. However, as described through linear stability analyses, the activity-induced instability is a generic mechanism that is also expected for extensile activities corresponding, for example, to stress generation due to cell division events [8, 9].

Eqs. (1), (3) and (10) are solved using a hybrid lattice-Boltzmann method [10]. Unless otherwise specified, the dynamical numerical parameters that we use are $\rho = 40$, $\eta = 40/6$, $\Gamma = 0.3$, $\Gamma_\phi = 0.2$, $P = 0.33$, $\xi = 0.3$, $\mathcal{A} = 0.002$, $\mathcal{C} = 0.001$, $K = 0.001$ and we vary L between $L = 0.0005$ and $L = 0.008$ but in general take it to be $L = 0.002$. The domain size is chosen as 600×600 lattice sites, sufficiently larger than the size of the active ring (inner and outer radius of $a = 45$ and $b = 170$) to exclude any finite-size domain effects. The simulations start with zero velocity and the director field oriented along the radial direction, corresponding to the radially-elongated cells between the lumen and the outer interface of the organoid observed in the experiments [11].

II. ESTIMATE OF THE SURFACE TENSION

Working in two spatial dimensions, we define the surface tension as the free-energy cost corresponding to the interface, normalised by its length, according to

$$\sigma \equiv \frac{\mathcal{F}[\phi] - \mathcal{F}[\phi_{\text{bulk}}]}{L}. \tag{12}$$

Here $\phi(\boldsymbol{r}) \in [0,1]$ denotes a scalar field indicating the relative density of active material at a given point and ϕ_{bulk} describes a hypothetical profile of coexisting bulk phases

$$\phi_{\text{bulk}} = \begin{cases} 1 & x < h_0 \\ 0 & x > h_0, \end{cases} \tag{13}$$

with h_0 the position of the sharp interface between the two. Since our aim is to address the free-energetic cost as a result of the interface, we eliminate, per definition, the gradient in ϕ_{bulk} — hence the label hypothetical — and since the two bulk phases are at equilibrium, $\mathcal{F}[\phi_{\text{bulk}}] = 0$ must hold.

Thus, in order to compute the surface tension σ we must find the profile $\phi(\boldsymbol{r})$ that minimises the free-energy functional

$$\mathcal{F} = \int \mathrm{d}^2\boldsymbol{r}\;\; f\left(\boldsymbol{Q}, \nabla\boldsymbol{Q}, \phi, \nabla\phi\right), \tag{14}$$

with $f\left(\boldsymbol{Q}, \nabla\boldsymbol{Q}, \phi, \nabla\phi\right))$ as defined in eqs. (6)-(9). Here we impose $\phi(\boldsymbol{r})$ to be anti-symmetric about the interface to conserve its integrated value.

A proper minimisation of this free energy would have us minimise $\mathcal{F}$ with respect to both ϕ and $\boldsymbol{Q}$ simultaneously. However, we can significantly simplify the analysis by making the coupling $S \approx S_n\phi$ explicit and setting the equilibrium nematic order $S_n = 1$, as promoted by the f_Q term. This enables us to only regard minimisation with respect to ϕ.

This is equivalent to assuming that the magnitude of nematic order does not change appreciably within the active nematic film itself. With this in mind, functional minimisation of eq. (14) yields

$$(\mathcal{A}+\mathcal{C})\,\phi-3\,(\mathcal{A}+\mathcal{C})\,\phi^2+2\,(\mathcal{A}+\mathcal{C})\,\phi^3-\left(K+\frac{1}{2}L\right)\nabla^2\phi=0, \tag{15}$$

where we have neglected terms involving gradients of the director $\boldsymbol{n}$. These must necessarily arise from perturbations and so be of first order or higher in perturbation.

Subsequently introducing the characteristic length scale

$$l\equiv\sqrt{\frac{K+\frac{1}{2}L}{\mathcal{A}+\mathcal{C}}}$$

allows us to recast eq. 15 as

$$\phi-3\phi^2+2\phi^3=\phi'', \tag{16}$$

where primes indicate derivatives with respect to the auxiliary dimensionless coordinate

$$\chi=\frac{x-h_0}{l}.$$

We then multiply eq. 16 with ϕ' and integrate the result to yield

$$\frac{1}{2}\phi^2\,(1-\phi)^2=\frac{1}{2}\,(\phi')^2+C_1. \tag{17}$$

Noting that $\phi=1$, $\phi'=0$ must hold at $\chi\to-\infty$ and $\phi=0$, $\phi'=0$ must hold at $\chi\to+\infty$, we identify $C_1=0$. We stress that taking these limits to $\pm\infty$ only holds by virtue of assuming a small interface length l.

Following this, we integrate the remaining terms to arrive at the form

$$\log\frac{\phi}{1-\phi}=\pm\chi+C_2. \tag{18}$$

Here, we fix $C_2=0$ by demanding that, by symmetry, $\phi=\frac{1}{2}$ at $\chi=0$. We then identify the minus sign as the suitable choice in this region by checking the limits at $\chi\to\pm\infty$, resulting in the interface profile

$$\phi=\frac{1}{1+e^{+\chi}}. \tag{19}$$

Given that we have $f\,[\phi_{\text{bulk}}]=0$, the surface tension then follows as

$$\sigma=\int_{-\infty}^{+\infty}\mathrm{d}x\,f[\phi]=\frac{1}{6}\sqrt{\left(K+\frac{1}{2}L\right)(\mathcal{A}_{\text{binary}}+\mathcal{C}_{\text{LQ}})}. \tag{20}$$

This is the surface tension we use in the main text, the value of which we vary by varying L.

III. LINEAR STABILITY ANALYSIS: INVISCID MODEL ORGANOID

We motivate the (strong) hydrodynamic coupling assumed between the organoid surfaces in the main text by considering the stability of an inviscid model organoid. As in the main text, the organoid is modelled as an incompressible annulus with inner radius a and outer radius b, with sharp interfaces and identical surface tensions $\sigma_a=\sigma_b=\sigma$. We impose a pressure difference $p_{in}-p_{out}$ due to curvature between the inside and the outside of the annulus to ensure a finite radius can be supported in the quiescent state $\overline{v}_r=0$, $\overline{v}_\theta=0$ and $\overline{p}(r)=\int\mathrm{d}r\,\alpha S/r$.

Next, we apply the infinitesimal sinusoidal perturbations $a(t)=a_0+\delta_a(t)e^{in\theta}$ and $b(t)=b_0+\delta_b(t)e^{in\theta}$, yielding perturbatory corrections to the velocity and pressure fields, $v_r\,(r,t)=\overline{v}_r+R\,(r,t)\,e^{in\theta}$, $v_\theta\,(r,t)=\overline{v}_r+\Theta\,(r,t)\,e^{in\theta}$ and $p\,(r,t)=\overline{p}(r)+P\,(r,t)\,e^{in\theta}$. These perturbations evolve according to the Navier-Stokes eqs. (10), where we take into

account only the isotropic and active contributions to the stress tensor. Expanding to first order in perturbation, the dynamical equations read

$$\begin{cases} \partial_t R = -\frac{1}{\rho} P' + \frac{\alpha S}{\rho} \frac{n^2 - 1}{b - a} \left\{ \frac{b - r}{a^2} \delta_a + \frac{r - a}{b^2} \delta_b \right\} \\ \partial_t \Theta = -\frac{in}{\rho} \frac{P}{r} \\ R' + \frac{R}{r} + in \frac{\Theta}{r} = 0. \end{cases} \tag{21}$$

Here primes denote derivatives with respect to the radial coordinate r, and we presume the organoid is at constant and homogeneous density ρ, activity α and orientational order S. The last term on the first line indicates the active forces induced by the perturbed surfaces, which we assume decay linearly away from the surface.

We solve eq. (21) with the set of boundary conditions

$$\begin{cases} R(a,t) = \dot{\delta}_a(t) \\ R(b,t) = \dot{\delta}_b(t) \\ P(a,t) = -\frac{\sigma}{a^2} \left(n^2 - 1\right) \delta_a(t) \\ P(b,t) = \frac{\sigma}{b^2} \left(n^2 - 1\right) \delta_b(t), \end{cases} \tag{22}$$

imposing continuity and normal stress balance at the surfaces. Here the dot denotes a time derivative, and the tangent stress balance is automatically satisfied for the inviscid case.

Following a procedure similar to that of Dumbleton and Hermans [12], we recast eqs. (21) and (22) in the matrix form

$$\begin{aligned} \ddot{\delta}_a &= M_{11}\delta_a + M_{12}\delta_b, \\ \ddot{\delta}_b &= M_{21}\delta_a + M_{22}\delta_b, \end{aligned} \tag{23}$$

where the matrix elements obey

$$\begin{aligned} M_{11} =& -n \frac{\left[\sigma (b-a)\left(n^4 - 5n^2 + 4\right) - \alpha S a \left(n^2 (2a - b) + 4b - 2a\right)\right] \coth\left(n \log \frac{b}{a}\right)}{\rho a^3 (b-a)(n^2 - 4)} \\ &+ \frac{n\alpha S}{\rho} \frac{na\left(n^2 (b-a) - 4b + a\right) + \left(n^2 + 2\right) b^2 \operatorname{csch}\left(n \log \frac{b}{a}\right)}{a^3 (b-a)(n^2-4)}, \\ M_{12} =& -n \frac{\left[\sigma (b-a)\left(n^4 - 5n^2 + 4\right) + \alpha S b \left(n^2 (2b - a) + 4a - 2b\right)\right] \operatorname{csch}\left(n \log \frac{b}{a}\right)}{\rho a b^2 (b-a)(n^2 - 4)} \\ &+ \frac{n\alpha S a^2}{\rho} \frac{3n + \left(n^2 + 2\right) \coth\left(n \log \frac{b}{a}\right)}{a b^2 (b-a)(n^2-4)}, \\ M_{21} =& -n \frac{\left[\sigma (b-a)\left(n^4 - 5n^2 + 4\right) + \alpha S a \left(n^2 (2a - b) + 4b - 2a\right)\right] \operatorname{csch}\left(n \log \frac{b}{a}\right)}{\rho a^2 b (b-a)(n^2 - 4)} \\ &+ \frac{n\alpha S b^2}{\rho} \frac{-3n + \left(n^2 + 2\right) \coth\left(n \log \frac{b}{a}\right)}{a^2 b (b-a)(n^2-4)}, \\ M_{22} =& -n \frac{\left[\sigma (b-a)\left(n^4 - 5n^2 + 4\right) - \alpha S b \left(n^2 (2b - a) + 4a - 2b\right)\right] \coth\left(n \log \frac{b}{a}\right)}{\rho b^3 (b-a)(n^2 - 4)} \\ &+ \frac{n\alpha S}{\rho} \frac{nb\left(n^2 (b-a) + 4a - b\right) + \left(n^2 + 2\right) a^2 \operatorname{csch}\left(n \log \frac{b}{a}\right)}{b^3 (b-a)(n^2-4)}. \end{aligned} \tag{24}$$

The solutions of eq. (23) take the form

$$\begin{aligned} \delta_a &= M_{12} C_\alpha e^{\omega_\alpha t} \\ \delta_b &= \left(\omega_\alpha^2 - M_{11}\right) C_\alpha e^{\omega_\alpha t}, \end{aligned} \tag{25}$$

where each C_α denotes an integration constant and the growth rates ω_α are solutions of the equation

$$\omega^4 - (M_{11} + M_{22})\,\omega^2 + M_{11}M_{22} - M_{12}M_{21} = 0. \tag{26}$$

This indicates the organoid surfaces exhibit identical growth rates ω_α: hydrodynamic interactions couple the inner and outer surface, as assumed in the main text.

Finally, eq. (26) also enables us to compute the activity threshold required to produce unstable perturbations. To this end, we set $\omega \to 0$ and look for the lowest-order instabilities, $n = 2$. Solving for α gives

$$\alpha_* = \frac{\sigma}{2S}\left[\frac{\begin{array}{c}6\left(a^2+b^2\right)+2\left(a-b\right)\left(a+b\right)\left(3+3\cosh\left(2\log\frac{b}{a}\right)\right)\operatorname{csch}\left(2\log\frac{b}{a}\right)\\ +\left|10ab-6a^2-6b^2+6\left(b^2-a^2\right)\coth\left(2\log\frac{b}{a}\right)+6\left(b^2-a^2\right)\operatorname{csch}\left(2\log\frac{b}{a}\right)\right|\end{array}}{2ab\left(a-b\right)+2ab\left(a+b\right)\coth\left(2\log\frac{b}{a}\right)-2\left(a^3+b^3\right)\operatorname{csch}\left(2\log\frac{b}{a}\right)}\right]. \tag{27}$$

The dependence of the activity threshold on σ is expected: increasing the surface tension essentially increases the pressure stabilising perturbations, thus requiring a higher activity to drive them unstable. Similarly, increasing the nematic scalar order parameter S focuses active effects, effectively bolstering the driving force behind the instability; hence the inverse proportionality. Finally, one can check numerically that increasing the surface area enclosed by the organoid — over which the active forces arise — decreases the activity threshold. Increasing the radius of the organoid at constant surface area, however, increases the activity threshold since this smears out any gradients driving the instability.

IV. LINEAR STABILITY ANALYSIS: OVERDAMPED MODEL ORGANOID

In the main text we present an extension of the above model that also includes viscous contributions. Operating in the limit of overdamped friction, the linearised Navier-Stokes equations read

$$\begin{cases}\partial_t R = -\frac{1}{\rho}P' + \frac{\alpha S}{\rho}\frac{n^2-1}{b-a}\left\{\frac{b-r}{a^2}\delta_a + \frac{r-a}{b^2}\delta_b\right\} - \chi R\\ \partial_t\Theta = -\frac{in}{\rho}\frac{P}{r} - \chi\Theta\\ R' + \frac{R}{r} + in\frac{\Theta}{r} = 0,\end{cases} \tag{28}$$

where we have replaced the viscous contributions with terms proportional to the overdamped friction coefficient χ.

The overdamped friction term in eq. (28) is usually motivated through an argument similar to Stokes' law [13]. In essence, one uses the full viscous stress tensor to derive an approximate expression for the friction in the dynamical equations. However, the same viscous stress tensor cannot be used to derive the corresponding stress balance boundary conditions. After all, such an approach would leave our system overdetermined, as we lowered the order of our equations by replacing a fully viscous description with an overdamped friction term.

Instead, we treat the overdamped friction as a body force (similar to the active forces), such that the boundary conditions (22) remain unchanged. Then, since we know from the above that the organoid surfaces exhibit identical growth rates, we probe for instabilities of the form $\propto e^{\omega t}$, and sequentially solve eq. (28). The dispersion relation reads

$$\omega^4 + \frac{2\chi}{\rho}\omega^3 + A\omega^2 + \chi B\omega + C = 0, \tag{29}$$

with

$$
\begin{aligned}
A =&\frac{1}{2a^3b^3\left(n^2-4\right)\rho^2(a-b)}\Bigg\{2ab(a-b)\left(-\alpha n^2\rho S\left(-n^2\left(a^2+b^2\right)+4a^2+3ab+4b^2\right)+a^2b^2\left(n^2-4\right)\chi^2\right)\\
&-2n\rho b^3\coth\left(n\log\left(\frac{a}{b}\right)\right)\Big[-a\alpha S\left(n^2(b-2a)+2a-4b\right)+\left(n^4-5n^2+4\right)\sigma(a-b)\Big]\\
&+2\alpha n\rho\,\mathrm{csch}\left(n\log\left(\frac{a}{b}\right)\right)\left(n^2+2\right)S\left(a^5+b^5\right)+n\rho a^3\mathrm{csch}\left(n\log\left(\frac{a}{b}\right)\right)\Big[-b\alpha S\left(2b\left(n^2-1\right)-a\left(n^2-4\right)\right)\\
&+\left(n^4-5n^2+4\right)\sigma(b-a)\Big]\sinh\left(n\log\left(\frac{a^2}{b^2}\right)\right)\mathrm{csch}\left(n\log\left(\frac{a}{b}\right)\right)\Bigg\},\\
B =&\frac{n}{2a^3b^3\left(n^2-4\right)\rho^2(a-b)}\Bigg\{2ab\alpha nS(a-b)\left(a^2\left(n^2-4\right)-3ab+b^2\left(n^2-4\right)\right)\\
&-2b^3\coth\left(n\log\left(\frac{a}{b}\right)\right)\Big[-a\alpha S\left(n^2(b-2a)+2a-4b\right)+\left(n^4-5n^2+4\right)\sigma(a-b)\Big]\\
&+2\alpha\left(n^2+2\right)S\left(a^5+b^5\right)\mathrm{csch}\left(n\log\left(\frac{a}{b}\right)\right)\\
&a^3\sinh\left(n\log\left(\frac{a^2}{b^2}\right)\right)\mathrm{csch}^2\left(n\log\left(\frac{a}{b}\right)\right)\Big[-b\alpha S\left(2b\left(n^2-1\right)-a\left(n^2-4\right)\right)+\left(n^4-5n^2+4\right)\sigma(b-a)\Big]\Bigg\},\\
C =&\frac{n^2\left(n^2-1\right)\mathrm{csch}^2\left(n\log\left(\frac{a}{b}\right)\right)}{2a^3b^3\left(n^2-4\right)\rho^2(a-b)}\Bigg\{-2\alpha\sigma S\left(a^2\left(n^2-1\right)-ab\left(n^2-4\right)+b^2\left(n^2-1\right)\right)-ab\alpha^2\left(n^2-2\right)S^2(a-b)\\
&+\left(n^4-5n^2+4\right)\sigma^2(b-a)+\Big[-2\alpha\sigma S\left(-n^2\left(a^2-ab+b^2\right)+a^2-4ab+b^2\right)+ab\alpha^2\left(n^2-2\right)S^2(a-b)\\
&+\left(n^4-5n^2+4\right)\sigma^2(a-b)\Big]\cosh\left(2n\log\left(\frac{a}{b}\right)\right)+\alpha nS(a+b)\sinh\left(n\log\left(\frac{a^2}{b^2}\right)\right)\left(-ab\alpha S-\left(n^2-1\right)\sigma(a-b)\right)\\
&-2\alpha nS\sinh\left(n\log\left(\frac{a}{b}\right)\right)\left(-\alpha S\left(a^3+b^3\right)+3\sigma(a-b)(a+b)\right)\Bigg\}.
\end{aligned}
\tag{30}
$$

One can check that eq. (29) reduces to eq. (26) in the limit $\chi \to 0$; this is because we assume identical boundary conditions for both.

---

[1] M. L. Blow, S. P. Thampi, and J. M. Yeomans, "Biphasic, lyotropic, active nematics," Physical review letters, vol. 113, no. 24, p. 248303, 2014.

[2] J. W. Cahn and J. E. Hilliard, "Free energy of a nonuniform system. i. interfacial free energy," The Journal of chemical physics, vol. 28, no. 2, pp. 258–267, 1958.

[3] M. Doi, Soft matter physics. Oxford University Press, 2013.

[4] A. N. Beris and B. J. Edwards, Thermodynamics of flowing systems: with internal microstructure. No. 36, Oxford University Press on Demand, 1994.

[5] L. Giomi, L. Mahadevan, B. Chakraborty, and M. Hagan, "Banding, excitability and chaos in active nematic suspensions," Nonlinearity, vol. 25, no. 8, p. 2245, 2012.

[6] A. Goriely, "Five ways to model active processes in elastic solids: active forces, active stresses, active strains, active fibers, and active metrics," Mechanics Research Communications, vol. 93, 2017.

[7] R. A. Simha and S. Ramaswamy, "Hydrodynamic fluctuations and instabilities in ordered suspensions of self-propelled particles," Physical review letters, vol. 89, no. 5, p. 058101, 2002.

[8] D. Volfson, S. Cookson, J. Hasty, and L. S. Tsimring, "Biomechanical ordering of dense cell populations," Proceedings of the National Academy of Sciences, vol. 105, no. 40, pp. 15346–15351, 2008.

[9] A. Doostmohammadi, S. P. Thampi, T. B. Saw, C. T. Lim, B. Ladoux, and J. M. Yeomans, "Celebrating soft matter's 10th anniversary: cell division: a source of active stress in cellular monolayers," Soft Matter, vol. 11, no. 37, pp. 7328–7336, 2015.

[10] D. Marenduzzo, E. Orlandini, M. Cates, and J. Yeomans, "Steady-state hydrodynamic instabilities of active liquid crystals: Hybrid lattice boltzmann simulations," Physical Review E, vol. 76, no. 3, p. 031921, 2007.

[11] E. Karzbrun, A. Kshirsagar, S. R. Cohen, J. H. Hanna, and O. Reiner, "Human brain organoids on a chip reveal the physics of folding," Nature Physics, vol. 14, p. 515, 2018.

[12] J. Dumbleton and J. Hermans, "Capillary stability of a hollow inviscid cylinder," The Physics of Fluids, vol. 13, no. 1, pp. 12–17, 1970.

[13] C. K. Batchelor and G. Batchelor, An introduction to fluid dynamics. Cambridge university press, 2000.
